# The longitudinal association between potential stressful life events and the risk of psychosocial problems in 3-year-old children

**DOI:** 10.3389/fpubh.2023.1100261

**Published:** 2023-03-21

**Authors:** Amy van Grieken, Jie Luo, Esther M. B. Horrevorts, Cathelijne L. Mieloo, Ingrid Kruizinga, Rienke Bannink, Hein Raat

**Affiliations:** ^1^Department of Public Health, Erasmus University Medical Center, Rotterdam, Netherlands; ^2^Department of Transforming Youth Care, The Hague University of Applied Sciences, The Hague, Netherlands; ^3^Center for Youth and Family Rijnmond, Rotterdam, Netherlands

**Keywords:** stressful life events, psychosocial problems, SDQ, preschool child, emotional and social development

## Abstract

**Background:**

Experiencing certain potentially stressful life events can impact psychosocial well-being among school-aged children and adolescents. This study aims to evaluate the association between life events occurring before age 2 and risk of psychosocial problems at 3 years of age.

**Methods:**

All parents invited for the regular well-child visit when their child was 2 years of age by the preventive Youth Health Care in the Rotterdam-Rijnmond area, the Netherlands, were invited to participate in this study. In total 2,305 parents completed the baseline questionnaire at child age 2-years; 1,540 parents completed the questionnaire at child age 3-years. The baseline questionnaire included a life events assessment (12 items), and tension caused by the event (range 0–3). At child age 3-years the questionnaire included the Strengths and Difficulties Questionnaire (SDQ) to assess risk of psychosocial problems. Logistic regression models were applied.

**Results:**

In the current study 48.5% of families experienced ≥1 life event before child age 2 years. Divorce and problems in the relationship between the parents received the highest perceived severity score [respectively 2.1 (*SD* = 0.8) and 2.0 (*SD* = 0.7)]. Children experiencing ≥1 event before the age of 2 years were at higher risk of psychosocial problems at 3 years of age, compared to children that had experienced no life event (1–2 events OR = 1.50, 95%CI: 1.09; 2.06, and >2 events *OR* = 2.55, 95%CI 1.64; 4.00, respectively). When life events caused high perceived levels of tension, there was also an association with an increased risk of psychosocial problems at age 3-years (*OR* = 2.03, 95%CI 1.43; 2.88).

**Conclusions:**

Approximately half of children in our study experienced a potential stressful life event before the age of 2 years. Results suggest an association between experiencing a life event and risk of psychosocial problems at child age 3-years. These findings emphasize the need for child health care professionals to pay attention to life events taking place in the life of young children in order to provide appropriate support.

## Introduction

Psychosocial problems, problems in psychosocial functioning, can be divided into three groups: internalizing/emotional problems (e.g., depressive feelings; anxiety), externalizing/behavioral problems (e.g., hyperactivity; aggressive behavior), and social problems (e.g., difficulties in making or keeping contact with peers) (1). The prevalence of psychosocial problems differs per country and per age group also depending on assessment methodology (2–5).

Psychosocial problems are associated with severe adverse outcomes, such as impaired social skills, lower academic results, substance abuse, delinquency and elevated suicide risk (5) and can be persistent over time (6). Already in 1998, Lavigne et al. (7) showed that children with a risk score on the Child Behavior CheckList (CBCL) at 2–3 years of age, had a higher risk for being diagnosed with a psychiatric disorder up to 8 years later. Moreover, the authors concluded that the family context is associated with the onset and maintenance of psychosocial problems in young children (7).

Stressful life events are events that a person appraises as threatening, which in turn triggers a behavioral and physiological response that might cause implications related to disease (8). The experience of stressful life events in childhood have been linked to increased risk of chronic illness later in life as well as increased mortality risk (9–11). Examples of life events are divorce, moving, death of a loved one, unemployment of parents, and family conflicts (10). Stressful life events might have a direct impact on child health through a behavioral or physiological response, while they might also have an indirect impact on the child *via* the family context. How well a family is able to deal with a stressful life event is depending on multiple factors such as coping skills, resources for support, other stressors in the family (11)). Besides that, each stressful life event can be experienced differently be each individual and family, e.g., relocation might cause stress and tension in one family but not in another. This subjective experienced tension of a stressful event might also be indicative of its impact on the family. Previous research, mainly among school-aged children and adolescents, has demonstrated an association between stressful life events and child anxiety (12), depression (13–16), alcohol abuse (17), and internalizing symptoms (18–20). The impact of life events in preschool children (i.e., between age 1–3 years) is not known, while it may provide insight for timely and adequate support to potentially minimize or reduce the consequences on child well-being in both short and long term.

This study aimed to evaluate the association between life events occurring before child age 2 years and risk of psychosocial problems at 3-years of age. We hypothesized that both the number of life events, as well as parent-rated high tension caused by the life event, were associated with the risk of psychosocial problems at child age 3-years. We explored whether the association between experiencing a life event and the risk of psychosocial problems was different by family social economic characteristics (10, 21, 22).

## Materials and methods

### Ethics statement

Parents received written information about the study and were free to refuse to participation or stop participation at any time. The Medical Ethical Committee of the Erasmus Medical Center Rotterdam declared that the Medical Research Involving Human Subject Act (Dutch abbreviation WMO) did not apply to the present study and, subsequently, permission was given to carry out the study and to publish the results in scientific journals (number MEC-2014-152). This study was conducted by following the guidelines proposed in the World Medical Association Declaration of Helsinki.

### Design and data collection

A longitudinal study design was applied, a baseline measure was performed at child age 2-years and a follow-up measure at child age 3-years. Data was collected by parent-reported questionnaires.

In the Netherlands, there is well-organized community-based preventive pediatric and family care, collectively known as the preventive Youth Health Care (YHC). Until the age of 18 years, each child in the Netherlands is invited by the YHC to come in for a periodic well-child visit, free of charge (23). Although participation is voluntary, the compliance rate in the first 18 months after childbirth goes up to 95% in some regions (23).

For the current study, all parents who were invited by the YHC professionals (i.e., youth health care physician or nurse) in the city of Rotterdam, the Netherlands, for a well-child visit when their child was 2 years of age (November 2014 and July 2015), were invited to participate in this study. The invitation for the study accompanied the invitation for the 2-year well-child visit at the YHC-center. An information leaflet, consent form and baseline questionnaire, was sent to the parents. The baseline questionnaire contained questions about socio-demographic characteristics, life events and psychosocial health of the child. If parents wanted to participate, they would leave the baseline questionnaire with the signed consent form at the YHC-center during the well-child visit and the YHC returned the forms to the researchers. In total, the researchers received 2,316 written informed consent forms from parents. The baseline questionnaire was completed by 2,305 parents. See Supplementary Figure 1 for the study flow chart.

Participating parents were sent a follow-up questionnaire by post or e-mail, containing questions about general and psychosocial health of the child when their child was 3 years old. At the 3-year time point, the questionnaire was returned by 1,540 (66.5%) parents. For the current study participants with data from twins (*n* = 17), other caregivers than parents (*n* = 32) and missing data on the SDQ at age 3-years were excluded, leaving a study population of 1,470 participants for analyses.

### Measurements

#### Life events

At child age 2-years, parents were asked if 12 life events had occurred in the past 2 years. These life events were based upon the Adverse Life Events Scale for children and adapted for parental-report (24): relocation of the family; relocation of someone close to the child; tensions at work of one of the parents that are felt at home; unemployment of one of the parents; financial problems; quarrels with neighbors/friends/acquaintances/family; problems within relationship of parents; divorce; victim of burglary or fire; physical health problems of someone close to the family; mental health problems of someone close to the family; death of someone close to the family. The correlation between the life events was considered low (Spearman's rho 0.39). If a particular life event had occurred, a score of 1 was assigned to the item. If a life event did not occur, a score of 0 was assigned. A total score was calculated by summing up the scores of the 12 life events, with a range from 0 to 12. Subsequently, following the total score distribution, three groups were created based on the total score of the life events: no life events, 1–2 life events or >2 life events.

#### Tension experienced from life events

If respondents indicated a life event had occurred, they were asked to specify to what extent this caused stress or tension, using a 3-point scale (1 = a little, 2 = somewhat, 3 = a lot). Supplementary Table 1 presents the frequency and average tension scores per individual life event.

We hypothesized that experiencing one or more high tension events may have an association with psychosocial health of the child. To be able to indicate the impact of experienced tension of a life event on psychosocial health a new variable was calculated. This variable ‘overall tension experienced from life events' groups data based on tension level reported. A group “no events” (participants who reported not to have experienced any life event, considered the reference group), a group “low” tension (participants of whom all experienced life events had a tension score of 1) and a group ‘high' tension (participants whom reported at least one experienced life event with a tension score of 2 or 3). See Supplementary Table 2 for a cross tabulation.

#### Risk of psychosocial problems at age 3-years

Risk of psychosocial problems at child age 3 years was assessed by the Strengths and Difficulties Questionnaire (SDQ) (25). The SDQ consists of 25 items with three response options (0 = not true/1 = somewhat true/2 = certainly true) and can be divided into 5 subscales: emotional symptoms, conduct problems, hyperactivity/inattention, peer relationship, and prosocial behavior. For the purpose of this study only the scales related to psychosocial problems were used. A summation score of the first four subscales was calculated to generate the Total Difficulties Score (range 0–40). If a child has a Total Difficulties Score of ≥9, the child is considered at-risk of psychosocial problems (26, 27). If a child has a summation score of ≥3 on the emotional symptoms subscale or ≥4 on the conduct problems subscale, the child is seen as at-risk of emotional and/or conduct problems (26, 27). Cut-off scores for at risk of psychosocial problems are equal for boys and girls. Cronbach's Alfa for the scales were: Total Difficulties Scale 0.75, Emotional Problems subscale 0.58, Conduct Problems subscale 0.58.

#### Other measurements

Various child, parental and family characteristics were assessed. For the child socio-demographic characteristics gender, age (in months), and ethnic background were assessed. Ethnic background was classified as Dutch (both parents are born in the Netherlands) or non-Dutch (at least one parent is born outside the Netherlands) following the definition of Statistical Netherlands (28). Risk of psychosocial problems at child age 2 years was assessed by the Brief Infant-Toddler Social and Emotional Assessment (BITSEA). The BITSEA is a parent-report questionnaire, consisting of 42 items, with three response options (0 = not true/rarely; 1 = somewhat true/sometimes; 2 = very true/often). The BITSEA is comprised of two scales, a Problem scale (31 items, focusing on social-emotional/behavioral problems such as aggression, and anxiety) and a Competence scale (11 items, focusing on social-emotional abilities such as empathy, and prosocial behavior). Responses can be summed for each scale. Cut-off scores for at risk of psychosocial problems are equal for boys and girls ≥14 for the Problem scale and ≤15 on the Competence scale (29, 30). A child was considered at risk of psychosocial problems at age 2-years if the child scored at-risk on either the BITSEA Problem scale, the Competence scale, or both.

For both mother and father, age (in years), ethnic background, and educational level were assessed. Educational level was categorized as low (primary education, lower secondary education), middle (higher secondary education, vocational education) or high (higher vocational education, university) (28). Ethnic background of the parent was classified as Dutch (the parents' parents, i.e., grandparents of the child, were born in the Netherlands) or non-Dutch (at least one of the parents' parents was born outside the Netherlands) following the definition of Statistical Netherlands (28).

At family level family structure was assessed; whether the child lived with both parents or with a single parent.

### Statistical analyzes

The characteristics of the sample are presented using descriptive statistics. Chi-square tests were used to indicate statistically significant differences between the children with and without risk of psychosocial problems at age 3 years (*p* < 0.05).

Two multivariable logistic regression models were applied to evaluate the association between a) life events before age 2 years (no life event, 1–2 life events, >2 life events) and b) tension experienced by the life events (no events, low, high) and at risk of psychosocial problems at age 3 years. Risk of psychosocial problems at age 3-years (yes/no) was assessed with the SDQ total score, the subscale emotional problems, and the subscale conduct problems. All models were corrected for risk of psychosocial problems at child age 2 years (yes/no). The following covariates were evaluated: child gender, ethnic background, maternal education level and family structure. Maternal education level was associated with the outcome, while family structure was associated with number and overall tension of life events; both were added as covariates in the final models (*p* < 0.05). Odds ratios (OR) and 95% confidence intervals (95% CI) are reported and were considered statistically significant if the *p-*value reached a level of <0.05. Cross sectional analyses of life events at age 2-years and risk of psychosocial problems at 2-years are presented in the Supplementary Table 3. The results using the number of life events continuously (i.e., total score by summing up whether each life event happened yes/no, range 1–12) are presented in Supplementary Tables 4, 5. The results of the regression analyses between individual life events and child psychosocial health at age 3 were added as Supplementary Table 6.

Interaction between life events (i.e., number of life events and overall tension experienced) and relevant covariates (i.e., child gender, ethnic background, family structure, maternal/paternal education level) was tested in the association with risk of psychosocial problems at age 3-years (i.e., SDQ total score). Interaction terms were added to the logistic regression models. After correction for multiple testing (0.10/14) interaction terms were considered statistically significant at p <0.007. No significant interaction terms were observed (see Supplementary Table 7). In addition a non-significant interaction term was observed between number of life events and tension experienced (Supplementary Table 8).

Non-response analyses using Chi-square tests and *t*-tests were performed to compare socio-demographic characteristics of parents and children participating in the follow-up assessment (*n* = 1,540) and participants who were lost-to-follow-up between the baseline and follow-up assessment (*n* = 765). Results are presented in Supplementary Table 9.

Analyses were conducted using SPSS version 25 (31).

## Results

### Non-response analysis

Respondents who were lost to follow-up (*n* = 765), were more often of non-Dutch ethnic background (child 41.4 vs. 19.8%; mother 49.4 vs. 24.5%; father 45.1 vs. 22.0%; all *p* < 0.001), the children were more often at-risk of psychosocial problems at 2-years of age (28.5 vs. 17.2%, *p* < 0.001), mother and father more often had a low educational level (13.8 vs. 6.4% and 19.1 vs. 11.3%, respectively for mother and father, both *p* < 0.001), and were more often single parent families (14.7 vs. 6.4%, *p* < 0.001) compared to the participants in the current study (see Supplementary Table 9).

### Sample characteristics

The sample consisted of 1,470 children of which 48.9% was a boy. The children were on average 24.5 (*SD* = 1.8) months old, and 80.2% of the children were of Dutch ethnic background. Of the mothers, 6.2% had a low educational level and 75.7% was of Dutch ethnic background. Of the fathers, 11.4% had a low educational level and 78.2% was of Dutch ethnic background. Of 6.0% of the children the family structure was single parent (Table 1).

**Table 1:** Characteristics of the study population.

	**Total sample (*n* = 1,470)**	**SDQ total difficulties score**		
		**Not at risk (*****n*** = **1,214)**	**At risk (*****n*** = **256)**	**P-value**
**Child characteristics**				
Age child in months, mean ± SD	24.5 ± 1.8	24.5 ± 1.9	24.4 ± 1.6	0.347
Gender, boy [n(%)]	714 (48.9)	579 (48.0)	135 (52.9)	0.156
Ethnic background, Dutch [n(%)]	1133 (80.2)	946 (80.7)	187 (77.9)	0.321
Psychosocial problems age 2-years, at risk [n(%)]^1^	246 (16.9)	155 (12.9)	91 (36.3)	**<0.001**
**Parent characteristics**				
Maternal ethnic background, Dutch [n(%)]	1,068 (75.7)	894 (76.3)	174 (72.5)	0.206
Maternal educational level [n(%)]				**0.001**
High	862 (59.8)	741 (62.1)	121 (48.8)	
Middle	489 (33.9)	382 (32.0)	107 (43.1)	
Low	90 (6.2)	70 (5.9)	20 (8.1)	
Paternal ethnic background, Dutch [n(%)]	1,103 (78.2)	914 (78.4)	189 (77.1)	0.668
Paternal educational level [n(%)]				
High	732 (52.2)	641 (55.1)	91 (37.9)	**<0.001**
Middle	511 (36.4)	396 (34.0)	115 (47.9)	
Low	160 (11.4)	126 (10.8)	34 (14.2)	
**Family characteristics**				
Family structure, single parent [n(%)]	86 (6.0)	66 (5.5)	20 (8.1)	0.121
Number of life events [n(%)]				**<0.001**
No life event	741 (51.6)	642 (53.9)	99 (40.1)	
1–2 life events	544 (37.9)	442 (37.1)	102 (41.3)	
>2 life events	152 (10.6)	106 (8.9)	46 (18.6)	
Overall experienced tension from life events [n(%)]^2^				**<0.001**
No event	738 (51.7)	640 (54.1)	98 (40.3)	
Low	349 (24.5)	286 (24.2)	63 (25.9)
High	340 (23.8)	258 (21.8)	82(33.7)

Number of missing: child gender = 10, child ethnic background=58, child psychosocial problem at age 2 years =13, maternal ethnic background = 59, maternal educational level = 29, paternal ethnic background=59, paternal educational level = 67, family structure = 29, number of life events = 33, tension caused by life event if life event happened = 40.

At-risk of psychosocial problems was measured by the Brief Infan t –Toddler Social and Emotional Assessment. Having an at risk score on BITSEA Problem or Competence scale, or both scales, was considered “at-risk.”

“No events” (participants who reported not to have experienced any life event, considered the reference group), a group “low” tension (participants of whom all experienced life events had a tension score of 1) and a group “high” tension (participants whom reported at least one experienced life event with a tension score of 2 or 3).

Significant p-values are printed bold.

### Life events

Table 1 shows that 37.9% of the families experienced 1–2 life events, and 10.6% experienced >2 life events at child aged 2-years. Of all families experiencing a life event (*n* = 689), 50.7% (*n* = 349) experienced low and 49.3% (*n* = 340) experienced high tension levels. A total of *n* = 1,305 life events was reported.

The life events most often experienced were the relocation of the family (16.8%), tensions at work of parent, felt at home (16.5%) and physical health problems of someone close to the family (14.8%). Children more often scored at risk of psychosocial problems (*p* < 0.05) compared to not at risk when the following life events were reported: tensions at work of one of the parents, financial problems, physical or mental health problems of someone close to the family, problems within relationship between parents, divorce (see Table 2).

**Table 2:** Life events and risk of psychosocial problems at age 3-years.

	**SDQ total difficulties score**			
	**Total yes**^a^ **n (%)**	**Not at risk n (%)**	**At risk n (%)**	**P-value**
Relocation of the family [37]	241 (16.8)	200 (16.8)	41 (16.7)	0.944
Relocation of someone close to the child [36]	11 (0.8)	10 (0.8)	1 (0.4)	0.701
Tensions at work of one of the parents, felt at home [53]	233 (16.5)	170 (14.5)	63 (25.7)	**<0.001**
Financial problems [44]	56 (3.9)	33 (2.8)	23 (9.5)	**<0.001**
Unemployment of one of the parents [41]	139 (9.7)	106 (9.0)	33 (13.5)	**0.030**
Quarrels with neighbors/friends/acquaintances/family [42]	44 (3.1)	32 (2.7)	12 (4.9)	0.068
Problems within relationship of the parents [39]	73 (5.1)	45 (3.8)	28 (11.5)	**<0.001**
Divorce [39]	31 (2.2)	21 (1.8)	10 (4.1)	**0.024**
Victim of fire or burglary [35]	20 (1.4)	16 (1.3)	4 (1.6)	0.764
Physical health problems of someone close to the family [61]	209 (14.8)	161(13.8)	48 (19.8)	**0.016**
Mental health problems of someone close to the family [46]	107 (7.5)	78 (6.6)	29 (11.9)	**0.004**
Death of someone close to the family [43]	141 (9.9)	110 (9.3)	31 (12.7)	0.104

[Missing N] indicates the missing data on SDQ Total Difficulties Score for this life event. *P*-values based on Chi-square test or Fisher's Exact test (when one cell has an expected count 0 <T <5), significant *p*-values are printed bold.

^a^The number of parents that reported this specific life event to have taken place. The total number of unique life events reported by *n* = 696 parents.

### Life events, overall experienced tension, and risk of psychosocial problems

Compared to children having not experienced any life event before the age of 2-years, having experienced 1–2 life events was associated with a significant higher Odds Ratio (OR) for an at risk score for psychosocial problems (*OR* = 1.50, 95%Confidence Interval (CI) = 1.09; 2.06), and emotional problems (*OR* = 2.08, 95%CI = 1.27; 3.42) at child age 3 years. Also, having experienced >2 life events was associated with a significant higher OR for an at risk score for psychosocial problems (*OR* = 2.55, 95%CI = 1.64; 4.00), emotional problems (*OR* = 3.16, 95%CI = 1.65; 6.02), and conduct problems (*OR* = 2.21, 95%CI = 1.20; 4.09).

An overall high level of tension experienced from life events was associated with a significant higher OR for an at risk score for psychosocial problems (*OR* = 2.03, 95%CI = 1.43; 2.88), emotional problems (*OR* = 3.57, 95%CI=2.16; 5.92) and conduct problems (*OR* = 1.81, 95%CI = 1.09; 2.99; Table 3).

**Table 3:** Associations between experiencing life events before age 2 years and the risk of psychosocial problems at child age 3 years.

	**SDQ total difficulties score**	**SDQ emotional problems**	**SDQ conduct problems**
	**OR (95% CI)**	**OR (95% CI)**	**OR (95% CI)**
**Number of life events**			
No life event	Ref	Ref	Ref
1–2 life events	1.50 (1.09–2.06)^*^	2.08 (1.27–3.42)^*^	1.46 (0.92–2.33)
>2 life events	2.55 (1.64–4.00)^**^	3.16 (1.65–6.02)^**^	2.21 (1.20–4.09)^*^
**Overall experienced tension from life events**			
No events	Ref	Ref	Ref
Low	1.37 (0.95–1.98)	1.18 (0.63–2.22)	1.43 (0.85–2.41)
High	2.03 (1.43–2.88)^**^	3.57 (2.16–5.92)^**^	1.81 (1.09–2.99)^*^

SDQ, Strengths and Difficulties Questionnaire; OR, Odds Ratio; CI, Confidence Interval.

Logistic regression model: number of life events as independent variable and adjusted for baseline risk of psychosocial problems, maternal educational levels and family structure. Total *n* = 1,383 because of missing data on predictor and covariates.

Logistic regression model: overall tension caused by life events as independent variable and adjusted for baseline risk of psychosocial problems, maternal educational levels and family structure. Total *n* = 1,376 because of missing data on predictor and covariates. ^*^p < 0.05 and ^**^p < 0.01.

## Discussion

This study presents the longitudinal associations between potential stressful life events and risk of psychosocial problems in 3-year-old children. In addition, the tension or stress perceived by the life event was related to risk of psychosocial problems of the child. Approximately half of the families reported one or more life events before the child was 2-years old and at least one life event that caused high levels of stress. Notably, having experienced one or more life events before child age 2 was associated with higher odds for the child to be at risk of psychosocial problems at 3 years of age. Moreover, having experienced one or more life events that were reported to result in high tension levels, higher odds for the child to be at risk for psychosocial problems were observed.

Our study adds to the literature by confirming the impact of life events happening in a family when the child is still very young. Namely, life events between child age 1 and 2-years old, can have an impact on the 3-year old child's psychosocial well-being. Thus far, these studies were performed among school-aged children and adolescents (12, 13, 18, 32). Also, the current findings suggest a trend (data not shown) that the risk of psychosocial problems, at the age of 3, increases as more life events are experienced or when life events have a higher impact on the family. This increase in risk has been reported in previous research in 6-year-olds (33), and 11–13 year-olds (22).

More research is needed to explore the pathways and mechanisms that cause these longer term effects on child psychosocial health. A behavioral and physiological pathway has been suggested to explain impact on health and well-being (8). In young children the impact on psychosocial health may also be impacted *via* the family environment. Such as the potential of the family (environment) to cope with the life event, including the ability to seek and receive help when needed (11). In general, it has been shown that having stress as a parent affects psychosocial well-being of the children (34). Coping skills, social support networks and many more related factors are related to how a parent can deal with the stress that can accompany life events (8). Therefore, life events may impact the child directly, as well as through the way parents are able to deal with the situation at hand (34, 35). Moreover, children develop over time, including their coping skills, which can help them deal with the situation better and/or ask for help earlier (36). In order to better understand the impact of life events among young children, research should use mixed methods combining both qualitative and quantitative data collection methods. This can help gain in-depth insight in how children and families experience, cope and seek help for life events.

Some life events appeared to be more ‘common' than others when the frequency of reporting was considered (Supplementary Table 1). Relocation of the family was a ‘common' life event among the study sample. Relocation of the family had an on average low tension rating. Furniss et al. (33) reported a negative association between the relocation (consisting of family relocated, and going to a new nursery school) and mental health assessed by the Child Behavior Check List. Since the study of Furniss et al. (12) was performed among older children, it could be argued that young children (2–3 years of age) are not impacted from moving from one place to another, while for a 6-year-old, it can have a severe impact, as they are just entering school and start making friends. Divorce on the other hand was a less ‘common' event, but with an average high tension rating. A meta-analysis of Amato (37) has suggested that divorce has no negative impact in very young children, but only in older, school-aged children. In our study the numbers in the analyses were too low to draw reliable conclusions on impact of individual events. In a study by Pruett et al. (38), divorce was associated with a negative impact on child (0–6 years) mental health, only when the divorce brought along parental conflicts or social adversities. With regard to particular life events and their impact, in our analyses the numbers were too low to draw reliable conclusions. We recommend future studies to examine the pathways that lead to impact on child health; taking into account the context in which events take place and are coped with. Meanwhile, for professionals working within pediatric prevention and care it is important to be aware of the impact of life events on the family and specifically the psychosocial health and well-being of young children. An open dialogue with parents is important in order to be able to detect issues timely and refer to specialized care if needed. Early support may prevent long term problems for the child as well as the other family members.

### Methodological considerations

Strengths of the study include the longitudinal design among a population-based sample of parents and their children. We were able to assess psychosocial well-being at age 2 and 3 years with well-validated instruments. Also, a broad range of life events was assessed in the questionnaire.

Parent-report by questionnaire was used to collect data. Self-report by questionnaire is at age 2-years hardly possible and interview methods are also challenging. Therefore, for the purpose of population-based studies among large community samples the use of parent-report questionnaires can be considered best practice. Life events were assessed at child age 2-years only, we were not able to correct for the time that has passed since the life event happened, or events taking place between age 2 and 3 years. Life events were categorized in three groups. Other ways to calculate life events experience and impact are possible, as we also explored using the total number of life events experienced. However, due to the distribution of the frequencies the current approach was considered most appropriate. Future studies with larger sample size and therewith potentially higher numbers of life events experienced may explore the linear association further. Also, larger studies may have the opportunity to study individual life events and psychosocial health impact. This could have resulted in over- or underestimation of the associations observed. The non-response analyses showed selective drop-out of study participants from baseline to follow-up and therefore results should be interpreted with caution.

## Conclusion

This study showed half of families with preschool children have experienced one or more life events. Having experienced one or more life events in the family before the child is 2-years old was associated with risk for psychosocial problems of the child at 3-years of age. Professionals working in with families and children should pay attention to the impact that a broad range of life events might have on the health and well-being of both children and the family.

## Data availability statement

The raw data supporting the conclusions of this article will be made available by the corresponding author upon reasonable request.

## Ethics statement

The Medical Ethical Committee of the Erasmus Medical Center Rotterdam has reviewed the research proposal and declared that the rules laid down in the Medical Research Involving Human Subjects Act (also known by its Dutch abbreviation WMO) did not apply to this research proposal (number MEC-2014-152). This study was conducted following the guidelines proposed in the World Medical Association Declaration of Helsinki. The parents provided written informed consent to participate in the study.

## Author contributions

HR obtained the funding. HR and AG managed the research. EH, CM, IK, and RB undertook data collection. AG, EH, and HR conceived the research described in this paper. JL analyzed the data. AG drafted the manuscript with input of JL and HR. All authors provided input in interpreting the data. All authors critically reviewed and approved the manuscript.
