# Supplementary material for: The longitudinal association between potential stressful life events and the risk of psychosocial problems in 3-year-old children
Source: Front Public Health. 2023 Mar 21;11:1100261. doi: 10.3389/fpubh.2023.1100261 (PMC10070685; doi:10.3389/fpubh.2023.1100261)
Supplement: Supplementary file 1 [file Data_Sheet_1.PDF]

Supplementary material

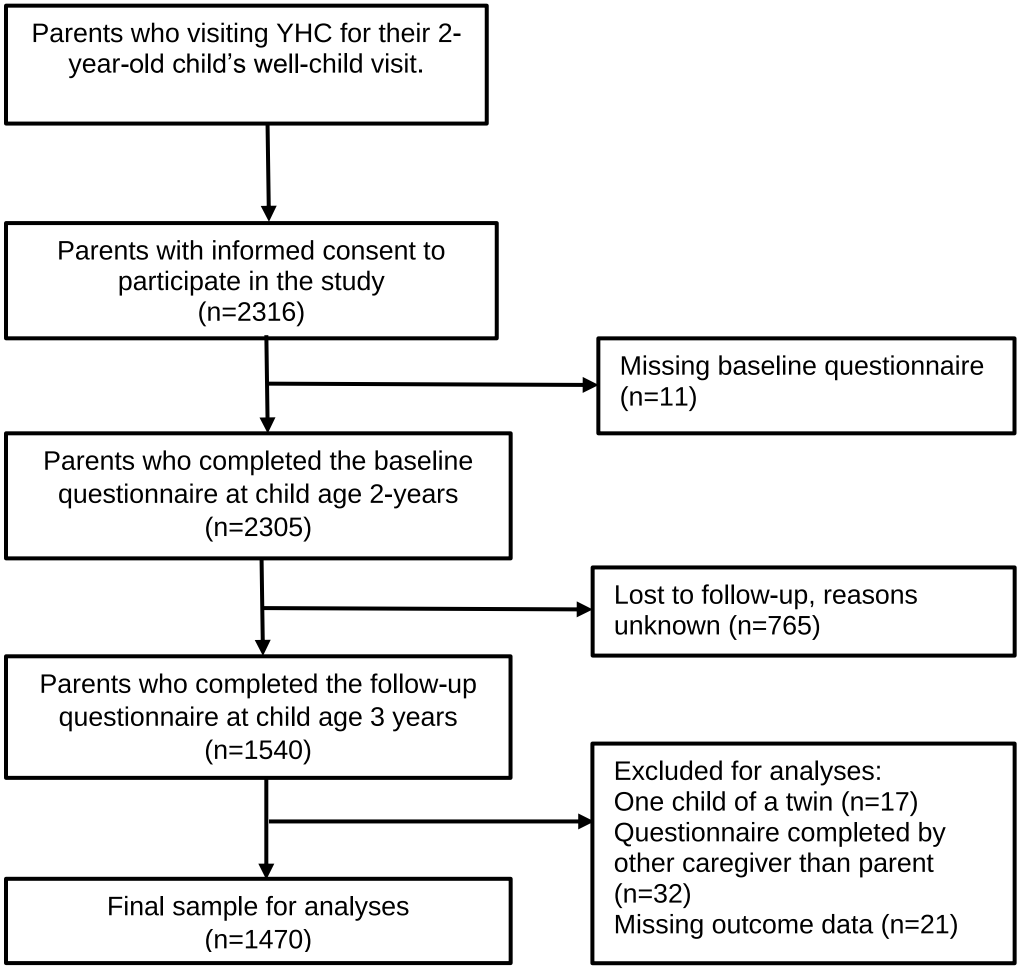

Figure 1. Population for analyses

Table S1. Tension score per life event (n=689).

		Tension score [7]			
	N[1]	1=A little (n)	2=Somewhat (n)	3=A lot (n)	Mean±SD
Relocation of the family	239	142	68	29	1.3±0.6
Relocation of someone close to the child	11	7	3	1	1.2±0.4
Tensions at work of one of the parents, felt at home	229	53	130	46	1.7±0.6
Financial problems	56	12	23	21	1.9±0.6
Quarrels with neighbours/friends/ acquaintances/family	44	8	22	14	1.8±0.6
Victim of fire or burglary	20	11	5	4	1.4±0.5
Physical health problems of someone close to the family	209	84	88	37	1.6±0.7
Mental health problems of someone close to the family	105	30	45	30	1.7±0.7
Death of someone close to the family	141	76	53	12	1.3±0.6
Problems within relationship of the parents	72	12	35	25	2.0±0.7
Divorce	31	8	11	12	2.1±0.8
Unemployment of one of the parents	138	65	46	27	1.4±0.6

Note: : SD=standard deviation.
[1] Frequency of life event reported among the n=689 participants

Table S2. Total number of life events and tension score frequencies (n=1470)

		Tension score [40]		
	Total N (%)	1=A little n (%)	2=Somewhat n (%)	3=A lot n (%)
No event	741 (51.8)	-	-	-
1-2 life events	537 (37.6	315 (90.3)	191 (74.0)	31 (37.8)
>2 life events	152 (10.6)	34 (9.7)	67 (26.0)	51 (62.2)
Total life events [33]	1430 (100.0)	349 (24.4)[1]	258 (18.0)[2]	82 (5.7)[2]

Note: [Missing N] indicates the missing data on the variable.
1Comprises the group of ‘low tension’ on the ‘Overall experienced tension from life events’ variable.
2Comprises the group of ‘high tension’ on the ‘Overall experienced tension from life events’ variable.

Table S3. Associations between experiencing a life event before age 2 years and being at risk for psychosocial problems at child age 2 years

	BITSEA Problem scale	BITSEA Competence scale	BITSEA[3]
	OR (95% CI)	OR (95% CI)	OR (95% CI)
Number of a life events[1]			
No life event	Ref	Ref	Ref
1-2 life events	1.86 (1.11-3.12)*	1.04 (0.73-1.49)	1.29 (0.94-1.76)
>2 life events	3.78 (2.04-7.02)**	1.11 (0.65-1.90)	1.86 (1.20-2.90)*
Overall experienced tension from life events [2]			
No events	Ref	Ref	Ref
Low	1.55 (0.85-2.83)	1.31 (0.89-1.93)	1.45 (1.02-2.05)*
High	3.04 (1.79-5.14)**	0.83 (0.54-1.29)	1.38(0.97-1.96)

Abbreviation: BITSEA=Brief Infant Toddler Social Emotional Assessment; OR=Odds Ratio; CI=Confidence Interval.
1 Logistic regression model: number of life events as independent variable (categorical) and adjusted for maternal educational level and family structure. Total n=1383 because of missing data on predictor and covariates.
2 Logistic regression model: overall experienced tension from life events as independent variable (categorical) and adjusted for maternal educational level and family structure. Total n=1376 because of missing data on predictor and covariates.
3 Having an at risk score on either or both the BITSEA Problem or Competence scale.
* p <0.05; ** p <0.01

Table S4. Associations between experiencing life events (continuous) before age 2 years and the risk of psychosocial problems at child age 3 years.

	SDQ Total Difficulties Score	SDQ Emotional problems	SDQ Conduct problems
	OR (95% CI)	OR (95% CI)	OR (95% CI)
Number of life events[1]	1.31 (1.17-1.46) **	1.38 (1.19-1.59) **	1.22 (1.06-1.41) *

Abbreviation: SDQ=Strengths and Difficulties Questionnaire; OR=Odds Ratio; CI=Confidence Interval..
1 Logistic regression model: number of life events as independent variable (as continuous variable) and adjusted for baseline risk of psychosocial problems, maternal educational levels and family structure. Total n=1383 because of missing data on predictor and covariates.
* p <0.05; ** p <0.01

Table S5. Associations between experiencing life events before age 2 years (continuous) and the risk of psychosocial problems at child age 2 years.

	SDQ Total Difficulties Score	SDQ Emotional problems	SDQ Conduct problems
	OR (95% CI)	OR (95% CI)	OR (95% CI)
Number of life events[1]	1.45 (1.26-1.67) **	1.02 (0.89-1.16)	1.19 (1.07-1.33)*

Abbreviation: SDQ=Strengths and Difficulties Questionnaire; OR=Odds Ratio; CI=Confidence Interval..
1 Logistic regression model: number of life events as independent variable (as continuous variable) and adjusted for baseline risk of psychosocial problems, maternal educational levels and family structure. Total n=1383 because of missing data on predictor and covariates.
* p <0.05; ** p <0.01

Table S6. Association between individual life events before age 2 years and the risk of psychosocial problems at child age 3-years.

**Life event**		SDQ Total Difficulties Score	SDQ Emotional problems	SDQ Conduct problems
	N[1]	OR (95% CI)[2]	OR (95% CI)[2]	OR (95% CI)[2]
Relocation of the family	241	1.01 (0.68-1.49)	1.60 (0.94-2.72)	0.89 (0.48-1.54)
Relocation of someone close to the child	11	0.56 (0.07-4.59)	1.79 (0.22-14.58)	1.49 (1.78-12.49)
Tensions at work of one of the parents, felt at home	233	**2.24 (1.57-3.20)****	**2.85 (1.76-4.61)****	1.59 (0.96-2.64)
Financial problems	56	**3.16 (1.70-5.88)****	**3.06 (1.40-6.66)****	**2.52 (1.15-5.54)***
Unemployment of one of the parents	139	1.41 (0.90-2.20)	1.27 (0.65-2.48)	1.54 (0.85-2.81)
Quarrels with neighbours/friends/ acquaintances/family	44	1.37 (0.65-2.91)	1.82 (0.68-4.88)	0.49 (0.11-2.12)
Problems within relationship of the parents	73	**3.89 (2.25-6.72)****	**2.59 (1.20-5.57)***	**2.35 (1.11-4.91)***
Divorce	31	2.40 (0.90-6.50)	1.42 (0.27-7.42)	1.35 (0.33-5.46)
Victim of fire or burglary	20	1.36 (0.43-4.31)	0.83 (0.11-6.34)	0.67 (0.09-5.18)
Physical health problems of someone close to the family	209	**1.53 (1.04-2.24)***	1.42 (0.80-2.52)	**1.94 (1.17-3.21)***
Mental health problems of someone close to the family	107	**1.70 (1.05-2.76)***	**1.78 (1.52-5.10)****	**2.52 (1.39-4.58)****
Death of someone close to the family	141	1.44 (0.91-2.27)	1.40 (0.72-2.73)	0.95 (0.46-1.96)

Abbreviation: SDQ=Strengths and Difficulties Questionnaire; OR=Odds Ratio; CI=Confidence Interval..
1 The number of parents that reported this specific life event to have taken place. The total number of unique life events reported by n=696 parents.

2 Logistic regression model: life events as independent variable and adjusted for baseline risk of psychosocial problems, maternal educational levels and family structure. Total n=1383 because of missing data on predictor and covariates.
* p <0.05; ** p <0.01

Table S7. Overview of interaction term significance in the models predicting the SDQ Total Difficulties Score.

	Number of life events	Overall experienced tension from life events
	P-value for interaction term	P-value for interaction term
Child gender	0.260	0.013
Child ethnic background	0.479	0.576
Maternal education level	0.322	0.450
Maternal ethnic background	0.028	0.523
Paternal education level	0.105	0.264
Paternal ethnic background	0.934	0.853
Family structure	0.389	0.406

Note: Correction for multiple testing was applied (p=0.10/ 14). Interaction term was considered significant at p<0.007. Significant interaction terms are presented in bold.

Table S8. Interaction between life events and experienced tension in the association with psychosocial problems at age 3-years.

Interaction	SDQ Total Difficulties Score	SDQ Emotional problems	SDQ Conduct problems
	P-value for interaction term	P-value for interaction term	P-value for interaction term
Number of events * tension	0.814	0.806	0.570

Abbreviation: SDQ=Strengths and Difficulties Questionnaire; OR=Odds Ratio; CI=Confidence Interval..

Table S9. Non-response analysis

	Response to follow-up		P-value
	No (n=765)	Yes (n=1540)	
Gender, boy [n(%)]	401 (52.7)	758 (49.5)	0.349
Child ethnic background, non-Dutch [n(%)]	289 (41.4)	286 (19.8)	<0.001
Maternal ethnic background, non-Dutch [n(%)]	348 (49.4)	355 (24.5)	<0.001
Paternal ethnic background, non-Dutch [n(%)]	311 (45.1)	319 (22.0)	<0.001
Maternal educational level, low [n(%)]	98(13.8)	94 (6.4)	<0.001
Paternal educational level, low [n(%)]	128 (19.1)	163 (11.3)	<0.001
Family structure, single parent [n(%)]	107 (14.7)	96 (6.4)	<0.001
Psychosocial problems age 2-years, at risk [n(%)][1]	214 (28.5)	263 (17.2)	<0.001

Note. Number of missing: child gender=16, child ethnic background=165, maternal ethnic background=163, paternal ethnic background=179, maternal educational level=132, paternal educational level=207, family structure=80, child psychosocial problem at age 2 years=39.
